# *Wolbachia* infection status and molecular diversity in the species of tribe Tagiadini Mabille, 1878 (Lepidoptera: Hesperiidae) collected in China

**DOI:** 10.1002/ece3.11279

**Published:** 2024-04-16

**Authors:** Xiaoying Wei, Jianqing Zhu, Ary A. Hoffmann, Jiqin Jia, Mengqi Xiao, Feiyu Duan, Yimin Zhang, Huimin Zhong, Jingyan Ge, Weidong Yu, Lei Zhang, Weibin Jiang

**Affiliations:** ^1^ College of Life Sciences Shanghai Normal University Shanghai China; ^2^ Shanghai Zoological Park Shanghai China; ^3^ School of BioSciences, Bio21 Institute The University of Melbourne Parkville Victoria Australia; ^4^ Shanghai No. 3 Girl's High School Shanghai China; ^5^ College of Continuing Education Shanghai Normal University Shanghai China

**Keywords:** horizontal transmission, mito‐nuclear discordance, recombination, Tagiadini, *Wolbachia*

## Abstract

*Wolbachia*, one of the most ubiquitous heritable symbionts in lepidopteran insects, can cause mitochondrial introgression in related host species. We recently found mito‐nuclear discordance in the Lepidopteran tribe Tagiadini Mabille 1878 from which *Wolbachia* has not been reported. In this study, we found that 13 of the 46 species of Tagiadini species tested were positive for *Wolbachia.* Overall, 14% (15/110) of Tagiadini specimens were infected with *Wolbachia* and nine new STs were found from 15 isolates. A co‐phylogenetic comparison, divergence time estimation and *Wolbachia* recombination analysis revealed that mito‐nuclear discordance in Tagiadini species is not mediated by *Wolbachia*, but *Wolbachia* acquisition in Tagiadini appears to have occurred mainly through horizontal transmission rather than codivergence.

## Graphical abstract

A co‐phylogenetic comparison, divergence time estimation and Wolbachia recombination analysis revealed that mito‐nuclear discordance in Tagiadini species is not mediated by Wolbachia, but Wolbachia acquisition in Tagiadini appears to have occurred mainly through horizontal transmission rather than codivergence. An investigation of the molecular diversity of Wolbachia in Lepidoptera confirmed horizontal transmission as a common phenomenon at the inter‐specific, inter‐generic and inter‐familial levels, without discernible geographic patterns.

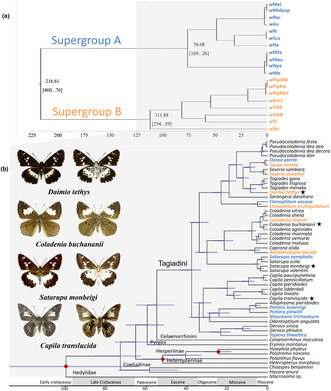

## 1 INTRODUCTION

*Wolbachia* bacteria, inherited maternally through their hosts, belong to alpha‐proteobacteria class and live in eukaryotic cells, especially in insects and some nematodes (Hoffmann, 2020). The supergroup designations are routinely used to describe the major phylogenetic subdivisions of this bacterial group and the multilocus sequence typing (MLST) scheme is a universal genotyping tool for *Wolbachia* (Baldo, Dunning Hotopp, et al., 2006), whereas full genome sequencing may be required to further establish relationships among *Wolbachia* strains (Conner et al., 2017; Cooper et al., 2019). More efforts in screening arthropods and genotyping *Wolbachia* are needed to better understand the ecology, evolution and establishment of *Wolbachia* symbiosis (Twort et al., 2022).

*Wolbachia* is commonly found in lepidopteran insects including moths and butterflies, and is associated only with supergroups A and B (Ahmed et al., 2016; Ahmed, Araujo‐Jnr, et al., 2015; Boonsit et al., 2023; Hamm et al., 2014; Ilinsky & Kosterin, 2017; Tagami & Miura, 2004; Twort et al., 2022). The proportion of infected Lepidoptera species at a non‐negligible frequency is estimated to be 80% and a quarter to a third of individuals appear to be infected with *Wolbachia* (Ahmed, Araujo‐Jnr, et al., 2015). *Wolbachia* requires transmission through the matriline (Bordenstein et al., 2009) and environments (Duplouy et al., 2020; Li et al., 2017; Sintupachee et al., 2006). They can affect the sexual reproduction of lepidopteran hosts, such as cytoplasmic incompatibility (CI) in *Hypolimnas bolina* and *Polygonia calbum* (Hornett et al., 2006; Kodandaramaiah et al., 2013), male killing (MK) in *Acraea encedon* and *Ostrinia* moths (Dyson et al., 2002; Fukui et al., 2015), feminization induction (FI) in *Eurema hecabe* (Kageyama et al., 2008) and parthenogenesis in *Tuta absoluta* (Erasmus et al., 2022). The *Wolbachia* can also affect various life‐history traits and impact on lepidopteran host biology, such as effects on longevity of *Talicada nyseus* (Ankola et al., 2013), fecundity of *Ectropis grisescens* (Zhang et al., 2021) and resistance to insecticides in *Chilo suppressalis* (Lei et al., 2020). Additionally, *Wolbachia* may cause mitochondrial introgression between sibling species or a selective sweep through a population, and drive a mito‐nuclear discordance between the host mitochondrial and nuclear phylogenies (Jiang et al., 2018; Narita et al., 2006; Toews & Brelsford, 2012; Zhu et al., 2023).

**FIGURE 1:**
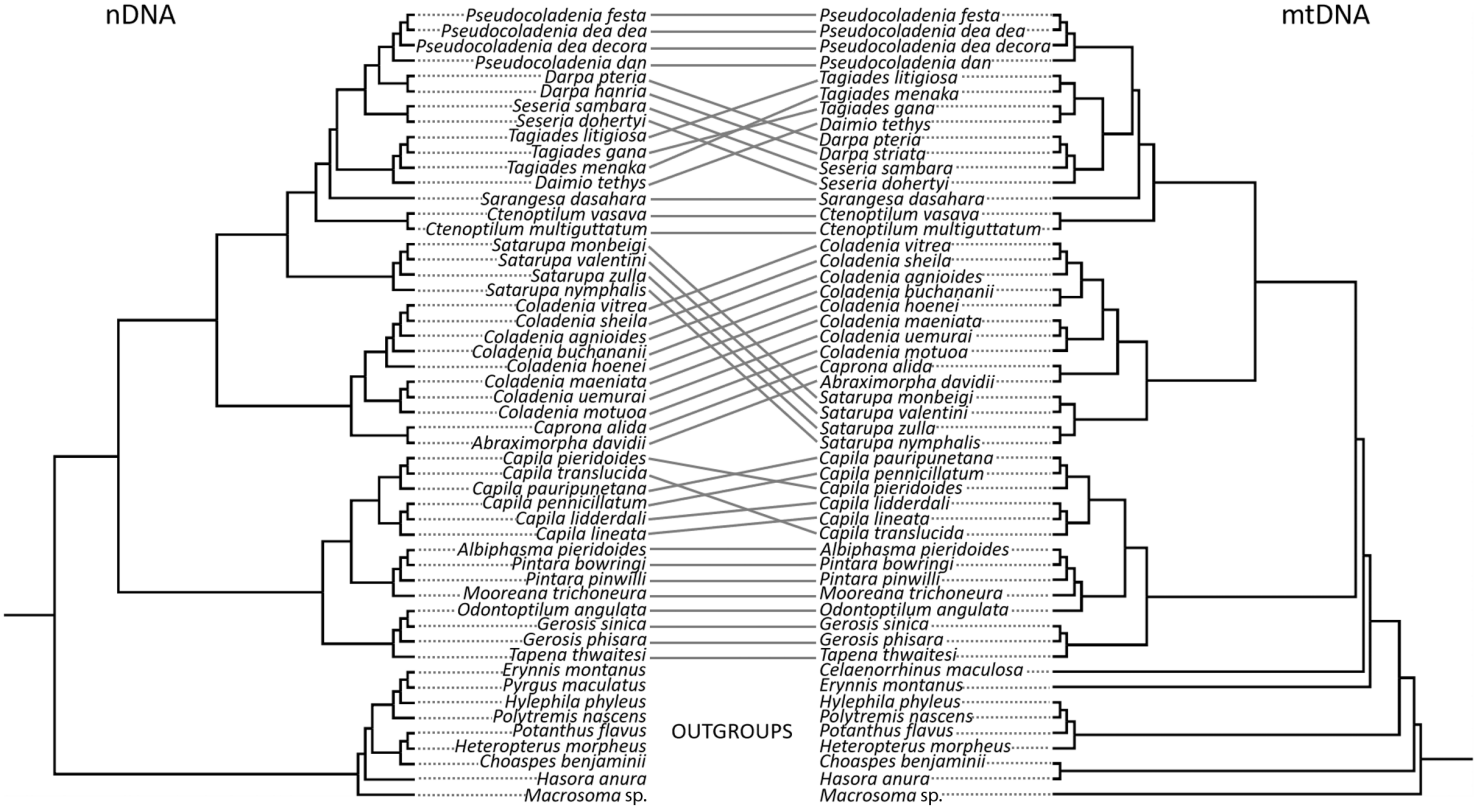
Cophylogenetic analysis of Tagiadini based on mtDNA (left) and corresponding nDNA (right). Connecting lines highlight the same species. Note the discordance in some parts of the phylogeny.

The Tagiadini Mabille 1878 tribe (c. 160 species) of Hesperiidae is one of the most species‐rich tribe within the subfamily Pyrginae (c. 650 species) (Evans, 1949; Ma, 2019). Our previous study (Shen et al., 2022) described the phylogenetic relationship of the tribe in China based on concatenated mitochondrial and nuclear genes and also noted a mito‐nuclear discordance in the molecular phylogeny of the Tagiadini (Figure 1). The discordance may be caused by species misidentification, incomplete lineage sorting (ILS), pseudogenes, introgressive hybridization and/or *Wolbachia* infection (Funk & Omland, 2003; Jiang et al., 2018; Toews & Brelsford, 2012). We excluded some of these such as species misidentification and pseudogenes in previous study (Shen et al., 2022). So far, there have been no systematic studies on Tagiadini members harbouring *Wolbachia* (Shen et al., 2022).

In the present study, we characterized the *Wolbachia* in this butterfly tribe by MLST genotyping, and compared a co‐phylogenetic relationship and the divergence time between *Wolbachia* and their Tagiadini hosts. Furthermore, we analysed the *Wolbachia* recombination and transmission patterns across Tagiadini species and revealed the potential effects of *Wolbachia* on variation and evolution of mtDNA. While *Wolbachia* has been investigated in many lepidopteran insects with a particular focus on infection status, sequence information, recombination or transmission patterns (Andersen et al., 2019; Arif et al., 2021; Bereczki et al., 2015; Dincă et al., 2019; Gaunet et al., 2019; Gislaine et al., 2018; Hinojosa et al., 2019; Jiang et al., 2014, 2016, 2018; Silva‐Brandão et al., 2021; Śliwińska et al., 2019; Yudina et al., 2016; Zhang et al., 2021), there are few studies that focus on the inter‐specific level across a large area. This has led us to complete a comprehensive survey of *Wolbachia* across the Tagiadini.

## 2 MATERIALS AND METHODS

### 2.1 Samples collection, DNA extraction and *Wolbachia* MLST typing

**FIGURE 2:**
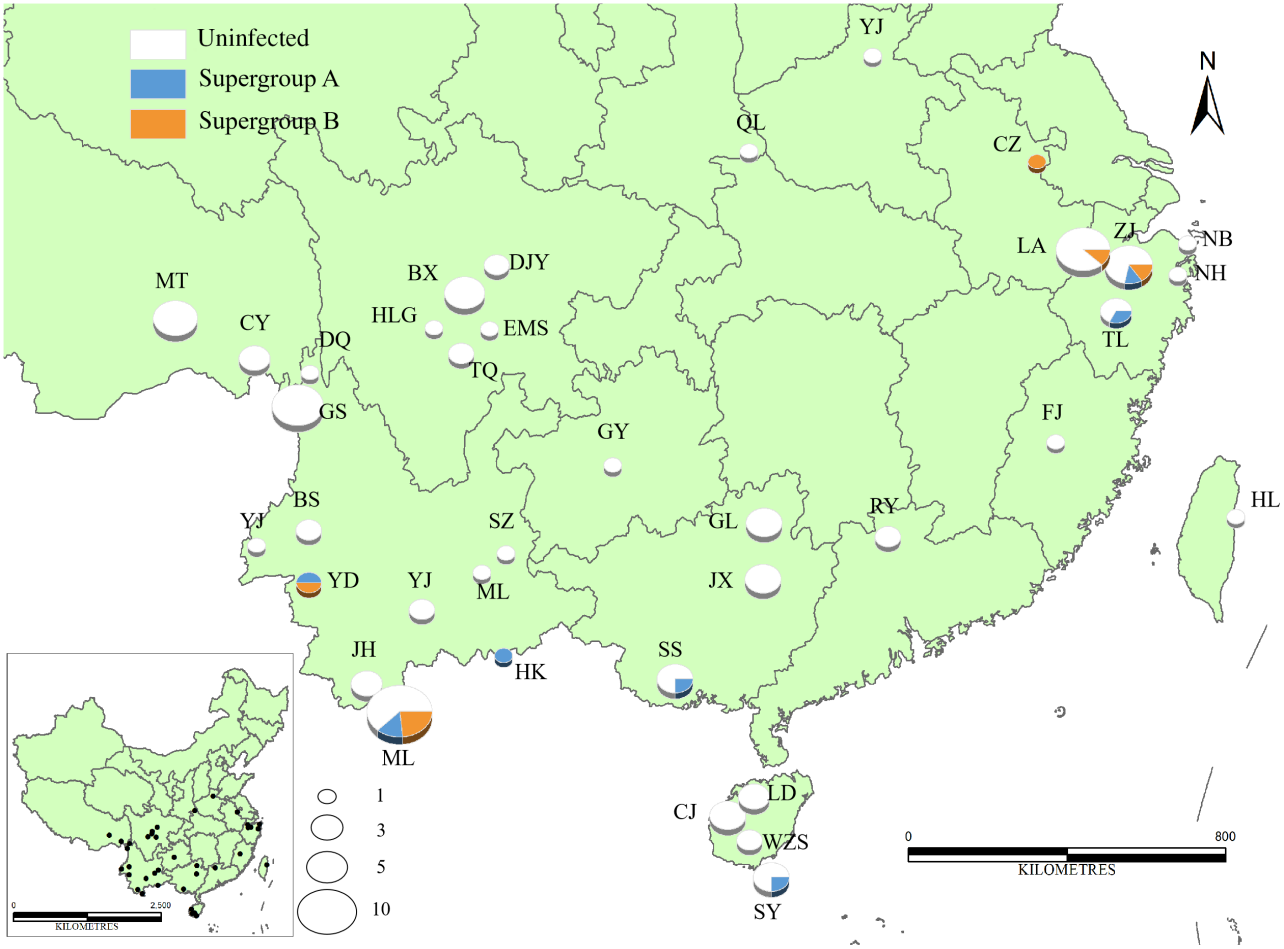
The distribution of Tagianidi specimens collected in China. The sizes of the circles are directly proportional to the number of individuals analysed. Three colour presents the different infection status (blue: infected with *Wolbachia* supergroup A; orange: infected with *Wolbachia* supergroup B; white: uninfected). The black dots refer to the location of collection sites and the letters are the abbreviation of place names. For full site names and other details, see Table S1.

We collected a total of 110 Tagiadini specimens representing 18 genera and 46 species from 38 local regions in China across the last 15 years (Figure 2 and Table S1). All specimens were caught in the field and preserved following dehydration in small envelopes. When the samples were sent to our laboratory, the abdomens of the butterflies were cut and preserved in 100% ethanol, which was subsequently held at −20°C until DNA extraction for molecular analysis. The preliminary species‐level identification was based on traditional morphological characteristics and molecular markers (Shen et al., 2022). The DNA was extracted from the entire adult butterfly abdomens using a QIAamp DNA Mini kit (Qiagen, Hilden, Germany) following the manufacturer's instructions.

To screen for the presence of *Wolbachia*, the *wsp* and *ftsZ* loci were amplified following the published protocols described by Zhou et al. (1998) (Baldo, Dunning Hotopp, et al., 2006) (Table S2). The characterization of *Wolbachia* genotypes was performed by sequencing multiple loci, including *gatB*, *coxA*, *hcpA*, *ftsZ* and *fbpA*, as recommended in the *Wolbachia* MLST database (http://pubmlst.org/wolbachia; Baldo, Dunning Hotopp, et al., 2006; Table S2).

### 2.2 Co‐phylogenetic analysis

For the molecular phylogenetic constructions of Tagiadini species (the concatenated mitochondrial and nuclear genes), we obtained the mitochondrial genes COI and COII, as well as three variable domains of nuclear DNA (D3 region of 28S rDNA, V4 and V7 regions of 18S rDNA) from GenBank (Table S1). The GTR + G model was selected as the best‐fit substitution model with PartitionFinder v2.1.1 (Lanfear et al., 2012) using the Bayesian Information Criterion (BIC). A maximum likelihood (ML) tree was constructed with the concatenated data using IQtree 1.4.2 (Nguyen et al., 2015). To assess nodal support, we performed 1000 ultrafast bootstrap replicates with UFBoot and an SH‐aLRT test with 1000 replicates (Hoang et al., 2018). The MLST database was searched for homologous sequences of newly obtained MLST and *wsp* genes. The *Wolbachia* MLST sequences were aligned with outgroups retrieved from the MLST database (Table S1) using Bioedit v. 7.0 (Hall, 1999). The NJ tree of *Wolbachia* MLST was constructed by Mega 7 (Kumar et al., 2016).

The genetic distance matrices of *Wolbachia* and hosts were compared with IBD using the Mantel test (Bohonak, 2002) performed on a pairwise node distance matrix of *Wolbachia* genotypes and host Tagiadini species to test for an association between matrices (Hall, 1999). Another test of phylogenetic congruence between butterflies and endosymbiont partners was undertaken with the Procrustean Approach to Cophylogeny (PACo; Balbuena et al., 2013). The analysis was performed in R with 100,000 permutations using packages VEGAN v.2.4.6 (Oksanen et al., 2017) and APE v.4.1 (Paradis et al., 2004).

### 2.3 Divergence time estimation and recombination analysis

We established a comparison between the divergence times of *Wolbachia* and the age of Tagiadini species divergence according to a molecular dating analysis of *Wolbachia* supergroups A and B (Gerth & Bleidorn, 2016). The divergence times of Tagiadini species were inferred with the relaxed‐clock molecular dating estimation by BEAST 1.5.2 (Excoffier et al., 2005). The analysis was performed using the HKY model of nucleotide substitution and assumed with the Yule speciation method. The best‐fit model was selected with PartitionFinder v2.1.1 (Lanfear et al., 2012) using the Bayesian Information Criterion (BIC). The age ranges provided by Chazot et al. (2019) were utilized to establish the calibration points for the divergence between Hesperiidae and Hedylidae (81–114 Mya), as well as the age ranges between Hesperiinae and Heteropterinae (35–55 Mya). Additionally, we applied a recently documented fossil hesperiid species, *Pamphilites abdita* Scudder, 1875 to constrain the minimum stem age of subfamily Hesperiinae to 25 Mya (de Jong, 2016). Chains were run for 50 million generations, with the first 20% discarded as burn‐in. The results were summarized with TRACER 1.5 (Fu & Li, 1993).

Gene recombination can interfere with and mislead the phylogenetic relationships of species. We detected recombination events with the MLST and *wsp* genes, to clarify whether horizontal transmission had occurred among these *Wolbachia* genotypes. In order to investigate recombination events among *Wolbachia* strains from Tagiadini species, each MLST gene and *wsp* gene were detected using RDP3 (Martin et al., 2010). Seven methods (RDP, GENECONV, BootScan, MaxChi, Chimaera, SiScan and 3Seq) in program RDP3 were chosen to identify the recombinant sequences and recombination breakpoints. The potential recombination events can be detected by any of the methods listed above. As recommended for this procedure, the breakpoint positions and recombinant sequences inferred from every potential recombination event were manually checked and adjusted following the phylogenetic and recombination signal analysis features available in RDP3. To visualize potential recombination events, ML trees for each MLST gene and *wsp* were constructed with three outgroups retrieved from the MLST database (Table S1) using IQtree 1.4.2 (Nguyen et al., 2015). They were checked for their supergroup clustering in ML trees. A potential recombination event can be found from inconsistencies between gene trees (Baldo, Bordenstein, et al., 2006).

## 3 RESULTS

### 3.1 Infection rates and diversity of *Wolbachia*

Tagiadini samples were screened for *Wolbachia* through the amplification of the *wsp* locus. Fifteen individuals in 13 species were positive for *Wolbachia* and were shown to be polymorphic for the infection despite limited sampling. The infection status and geographical distribution of each individual are listed in Figure 2 and Table S1. We characterized *Wolbachia* genotypes by MLST typing. The genotypes based on the MLST loci are denoted as *w*Ada, *w*Cho, *w*Cva1, *w*Cva2, *w*Cva3, *w*Dst, *w*Dpt, *w*Dte, *w*Mtr, *w*Ppi, *w*Pbo and *w*Sdo (Table 1). Seven genotypes belong to *Wolbachia* supergroup A and five genotypes belong to supergroup B. Three MLST genotypes (*w*Dst, *w*Mtr and *w*Ada) were identical to those available in *Wolbachia* MLST databases (https://pubmlst.org/wolbachia). *w*Dst was identical to ST‐41 belonging to *Wolbachia* supergroup B, *w*Mtr was identical to ST‐19 belonging to *Wolbachia* supergroup A and *w*Ada was identical to ST‐374 belonging to *Wolbachia* supergroup A. We found nine new STs from 15 isolates. ST‐41 and ST‐19 are the core genotypes of *Wolbachia* in lepidopteran hosts worldwide and detected in various Lepidopteran species (Ilinsky & Kosterin, 2017). Two *wsp* allels (OR479930 and OR479931) are new genotypes from *w*Dst (Table 1).

**TABLE 1:** *Wolbachia* MLST and *wsp* profiles for Tagiadini butterflies collected from China.

Sample ID	Species	*gatB*	*coxA*	*hcpA*	*ftsZ*	*fbpA*	*wsp*	Supergroup	Sequence types
SL004	*Abraximorpha davidii*	9	14	227	36	4	64	B	*w*Ada (ST374)
SL032	*Coladenia hoenei*	9	9	62	8	10	63	B	*w*Cho
SL049	*Ctenoptilum vasava*	37	37	62	3	4	114	A	*w*Cva1
SL050	*Ctenoptilum vasava*	7	37	7	3	8	114	A	*w*Cva2
SL051	*Ctenoptilum vasava*	37	37	93	3	40	114	A	*w*Cva3
SL053	*Ctenoptilum multiguttatum*	39	14	40	36	4	OR479930	B	*w*Dst (ST41)
SL057	*Darpa striata*	39	14	40	36	4	OR479931	B	*w*Dst (ST41)
SL060	*Darpa pteria*	111	6	7	3	8	442	A	*w*Dpt
SL062	*Daimio tethys*	39	14	93	36	4	116	B	*w*Dte
SL065	*Mooreana trichoneura*	7	6	7	3	8	31	A	*w*Mtr (ST19)
SL079	*Pintara pinwilli*	88	1	258	3	4	23	A	*w*Ppi
SL082	*Pintara bowringi*	7	6	93	3	8	114	A	*w*Pbo
SL084	*Satarupa nymphalis*	37	37	93	3	40	114	A	*w*Cva3
SL086	*Seseria doherty*	39	162	93	36	4	61	B	*w*Sdo
SL100	*Tapena thwaitesi*	37	37	93	3	40	114	A	*w*Cva3

### 3.2 Comparison of *Wolbachia* and Tagiadini phylogenies

**FIGURE 3:**
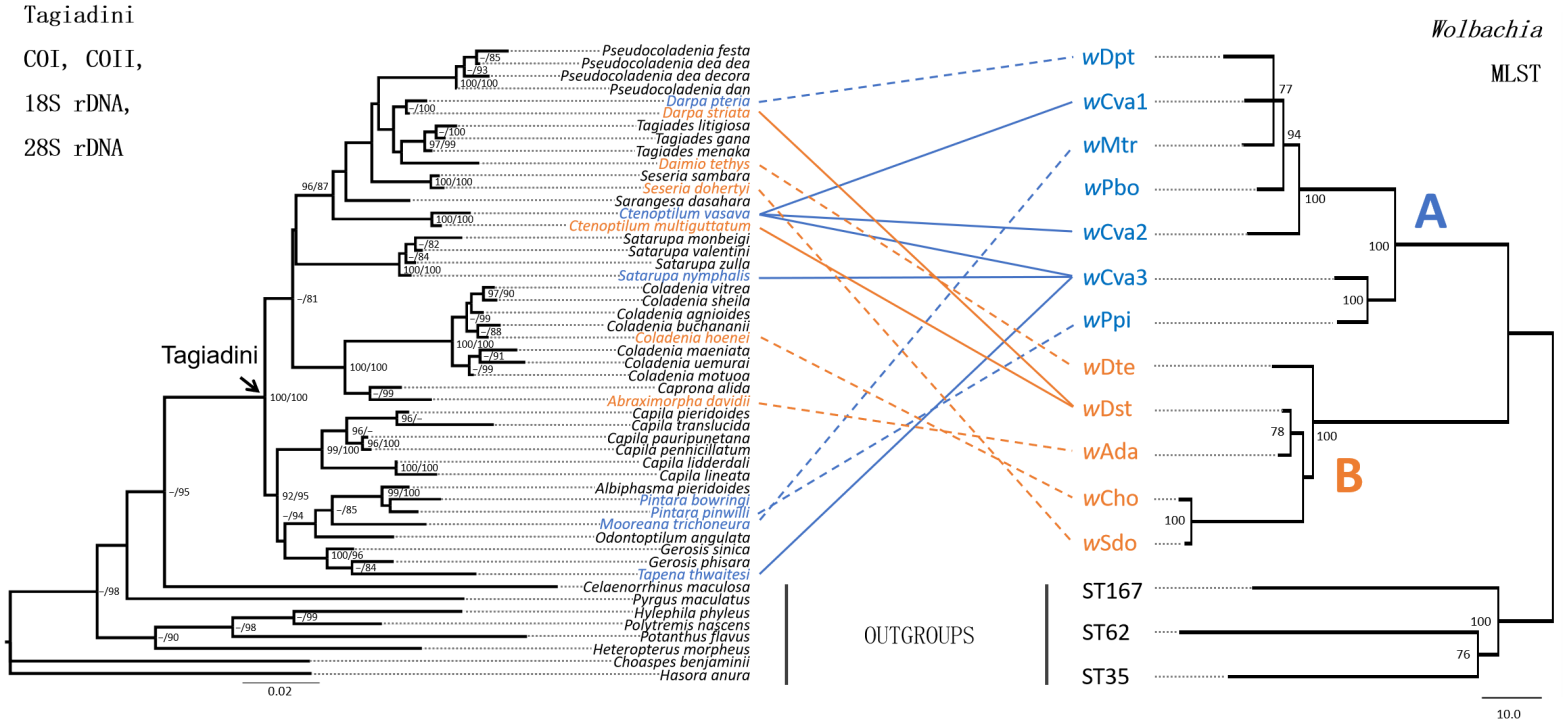
The tangled evolutionary relationships of Tagiadini when compared to their *Wolbachia* genotypes showing incongruence between host topology (left) and supergroup A and B *Wolbachia* topology (right). The orange solid lines point to a host switching event and the blue solid lines indicate a species harbouring different genotypes. The dotted lines connect the host species to the infected *Wolbachia* genotypes. Numbers beside nodes are IQTREE ultrafast bootstrap and SH‐aLRT values (left) and bootstrap values (right). The *Wolbachia* genotypes of Supergroups A are in blue and those of Supergroups B are in orange. Scale bars indicate the mean number of substitutions per site.

*Wolbachia* genotypes in this study were species specific except for a host switching event and a case of a species harbouring different genotypes. The genotype *w*Dst was shared by two host species (*Darpa striata* and *Ctenoptilum multiguttatum*) sympatric in Mengla, Yunnan Province, southwest China (Figure 2), while three *Ctenoptilum vasava* specimens harboured three genotypes respectively (*w*Cva1, *w*Cva2 and *w*Cva3). Additionally, the Co‐phylogenetic relationships were not congruent between *Wolbachia* genotypes and corresponding Tagiadini hosts (Figure 3). These suggested an important mode of transmission in Tagiadini *Wolbachia* was horizontal transmission. The Mantel test of genetic distances also provided no evidence for congruence between Tagiadini species and *Wolbachia* genotypes (*r* = .223, *p* = .624) in support of likely horizontal transmission. Similarly, PACo indicated no significant correlation between the phylogeny of Tagiadini and that of its endosymbiont (PACo m^2^ = 0.062, *p =* .217).

### 3.3 Divergence time estimation and recombination analysis

**FIGURE 4:**
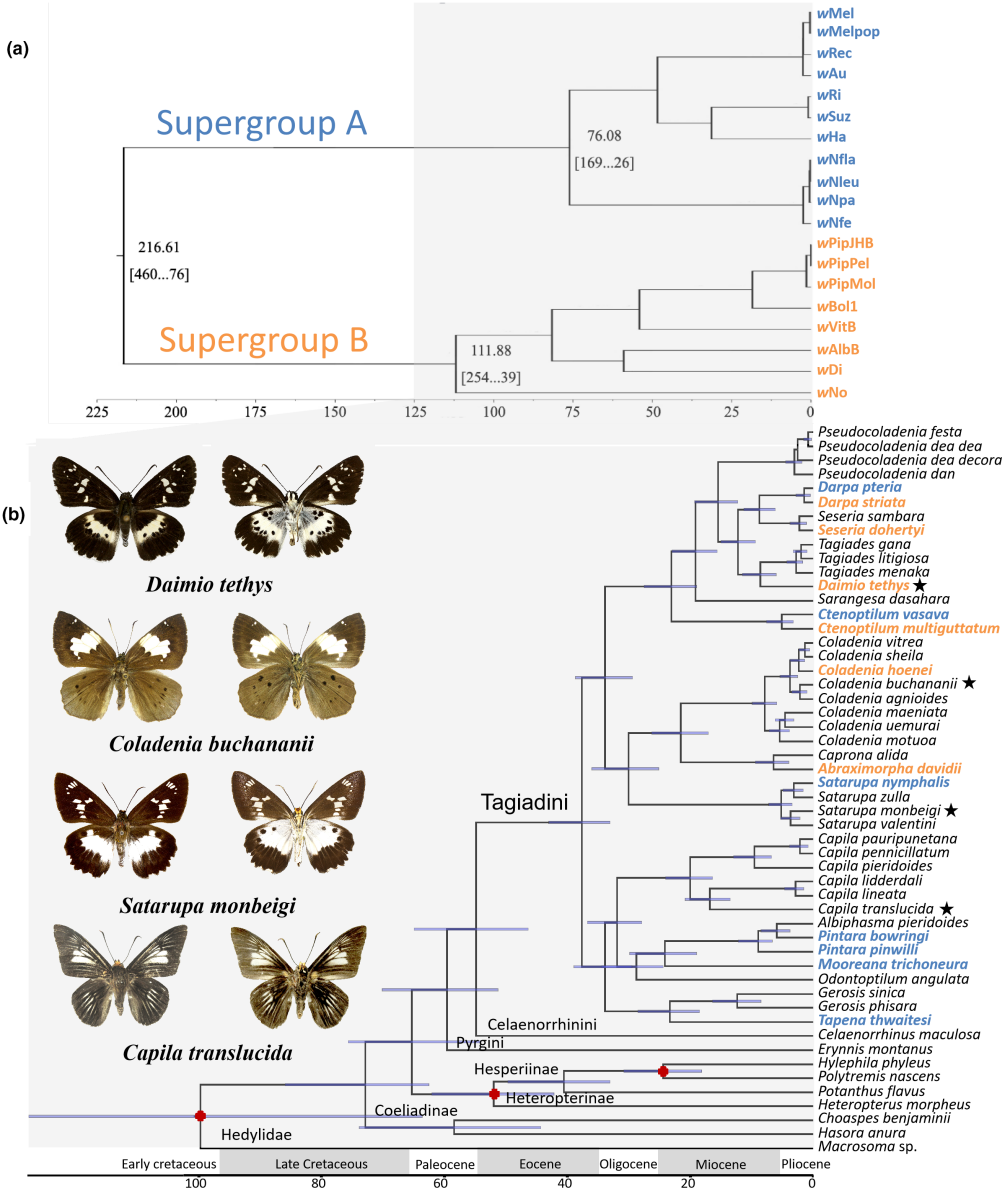
(a) Estimated divergence times of *Wolbachia* supergroups A and B based on Gerth and Bleidorn (2016), and (b) Bayesian Inference (BI) tree of mtDNA datasets for Tagiadini species using the uncorrelated lognormal relaxed clock in BEAST v1.5.2. Posterior probabilities of nodes are shown to the right of the node branch when higher than .95. The violet bars (b) indicate 95% highest posterior density interval (HPD) of the node ages. Asterisks indicate the illustrated species. The adults are depicted in dorsal (left) and ventral (right) aspects. The red dots are the calibration points for age ranges.

The relaxed clock molecular dating was implemented in BEAST and estimated with the divergence ages of the Tagiadini. The divergence time was compared between *Wolbachia* supergroups based on genomic data with data from Tagiadini in this study (Gerth & Bleidorn, 2016). We found the youngest divergence between species at 1.88 Mya (1.22–2.63, 95% HPD) and the oldest gap between *Odontoptilum angulata* and the other species at 28.75 Mya (39.5–24.84, 95% HPD) (Figure 4).

**FIGURE 5:**
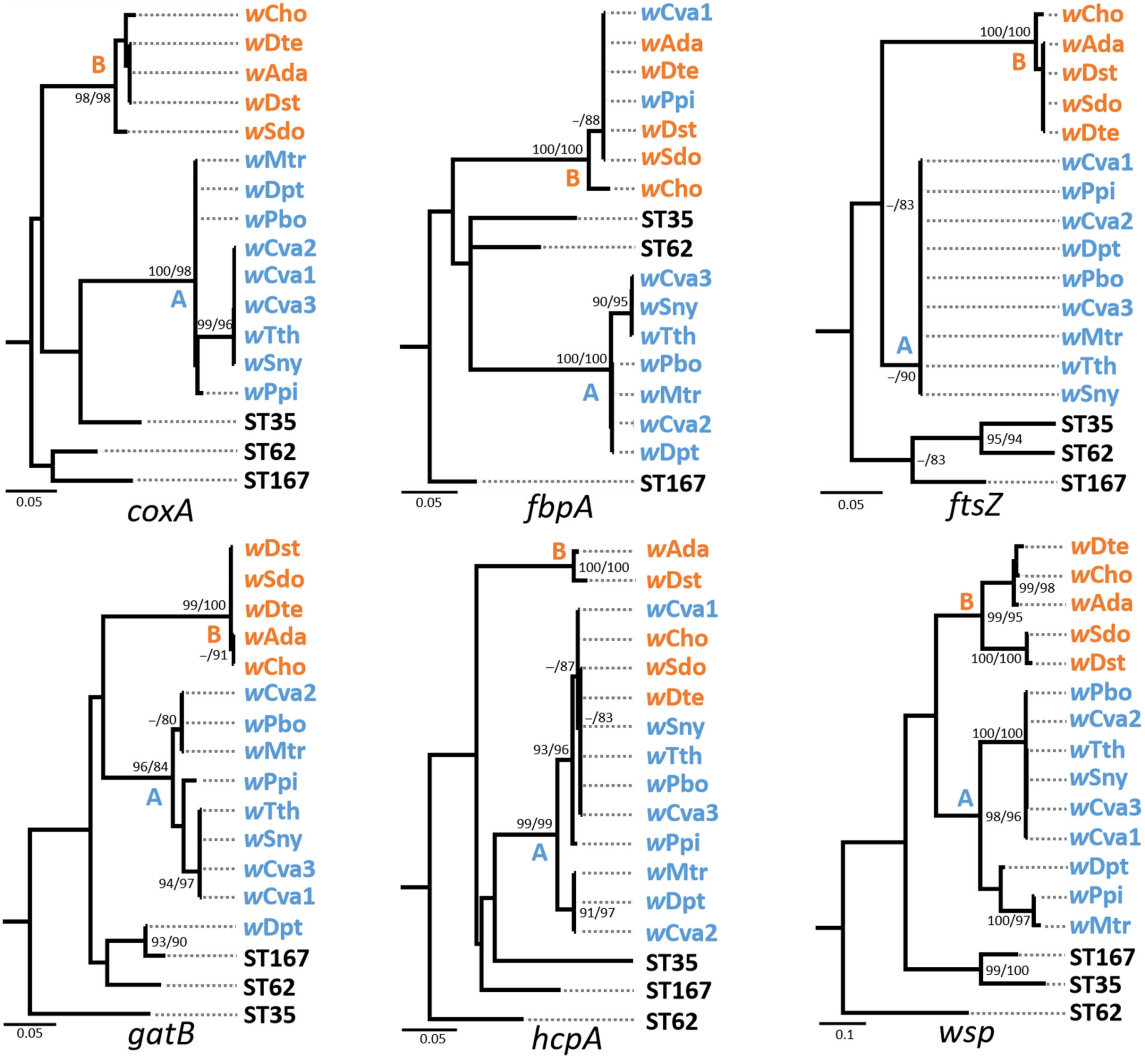
Maximum likelihood trees for each MLST gene and the wsp gene. Numbers beside nodes are IQTREE ultrafast bootstrap and SH‐aLRT values. The *Wolbachia* strains of supergroup A are in blue and those of supergroup B are in orange.

The recombination analysis found that the *wsp* sequence of the *w*Dpt from *Darpa pteria* is the recombinant between *Wolbachia w*Cva2 detected from *Ctenoptilum vasava* and *w*Mtr from *Mooreana trichoneura* (Figure S1). The polymorphic sites of the *Wolbachia wsp* alleles have a mosaic pattern, but not randomly distributed. The recombination events were confirmed with seven RDP3 algorithms (Table S3). ML trees for MLST genes and *wsp* gene were reconstructed with three outgroups separately (Figure 5 and Table S1). Five of the 12 *Wolbachia* genotypes (*w*Cva1, *w*Ppi, *w*Cho, *w*Sdo and *w*Dte) had inconsistent supergroup allocation among the five MLST gene trees as the potential recombination events.

## 4 DISCUSSION

We present the detailed analyses of *Wolbachia* in Tagiadini butterflies from China by screening. Thirteen species (28%, 13/46) tested positive for *Wolbachia* (from both supergroups A and B) and all of which are reported for the first time. The species incidence of *Wolbachia* in this taxon is low when compared with an overall estimate of around 80% for Lepidoptera species (Ahmed, Araujo‐Jnr, et al., 2015). Fourteen percent (15/110) of Tagiadini specimens were infected with *Wolbachia*. Larger sample sizes across the full geographical range of the Tagiadini tribe are needed to confirm these differences and more population samples are needed to establish the incidence of *Wolbachia* within infected species.

In the cophylogenetic analysis of Tagiadini based on mtDNA and corresponding nDNA (Figure S2), *Wolbachia* was not detected in some species with mito‐nuclear discordance (e.g. six species of genus *Capila*), while infected species with mito‐nuclear congruence were also detected (two species of genus *Ctenoptilum*, two species of genus *Pintara*, *Mooreana trichoneura* and *Tapena thwaitesi*). The remaining species infected with *Wolbachia* show mito‐nuclear discordance with closely related uninfected species. It may further complicate the interpretation. In this study, we only examined and explained the infection by present. Thus, there is not necessarily a direct association between mito‐nuclear discordance in Tagiadini species and the presence of *Wolbachia*. The other bacterial symbionts such as *Rickettsia*, *Cardinium* or *Spiroplasma* were likely responsible for this (Zakharov, 2015). Additionally, incomplete lineage sorting presumably can also lead to discordance between mtDNA and nDNA, but our restricted sample size and the few substitutions detected in the nuclear markers tested here make it difficult to use these data to reconstruct fine‐scale phylogenies.

The recombination analysis showed that *wsp* gene in *w*Dpt was an approximate intragenic recombination using RDP3 (Figure S1), suggesting a likely horizontal transmission occurred in the Tagiadini *Wolbachia* strains. The *wsp* gene shows a heterogeneous pattern of amino acid diversity characteristic marked by four distinct hypervariable regions (HVRs) interspaced by conserved strings of amino acids (CRs) (Baldo et al., 2005). Variants at each HVR have been frequently exchanged across bacterial strains, generating highly chimeric proteins (Baldo et al., 2005, 2010). The recombination events were also found in five *Wolbachia* genotypes (*w*Cva1, *w*Ppi, *w*Cho, *w*Sdo and *w*Dte) by checking MLST genes and *wsp* alleles for supergroup localization (Figure 5). The inconsistent supergroup allocation of alleles indicated that these genes had undergone independent evolutionary trajectories (Figure 5). This has also been observed in rice planthoppers (Zhang et al., 2013), spiders (Yang et al., 2021), parasitic wasps (Shaikevich & Romanov, 2023), fruit flies (Singh, 2019), moths and butterflies (Ilinsky & Kosterin, 2017).

Our results indicate that the phylogenetic relationships of Tagiadini hosts and corresponding *Wolbachia* genotypes were not congruent (Figure 3). Meanwhile, the correlation of genetic distances between *Wolbachia* genotypes and their Tagiadini hosts was not found by Mantel test and PACo, supporting the route of horizontal transmission of Tagiadini *Wolbachia*. The divergence age of *Wolbachia* was compared with that of their butterfly species (Figure 4). The divergence time between *Wolbachia* supergroups A and B was estimated over 200 Mya (Gerth & Bleidorn, 2016), while Tagiadini speciation events were dated between 1.88 and 28.75 Mya. This implies that the cocladogenesis is impossible between Tagiadini *Wolbachia* and their hosts because the *Wolbachia* genotypes have not been passed down from their common ancestor or transferred via hybridization events between the Tagiadini species. Two examples of likely horizontal transmission are provided by *w*Dst and *w*Cva3. *w*Dst is shared by Tagiadini species including *Darpa striata* and *Ctenoptilum multiguttatum*. These two butterflies are sympatric in Mengla, reflecting an opportunity for horizontal transmission (Figure 2). *w*Cva3 is shared by *Ctenoptilum vasava*, *Satarupa nymphalis* and *Tapena thwaitesi*, all collected from Yunnan province.

Although there is a growing number of studies indicating or proving horizontal transmission of *Wolbachia* among coleopteran hosts, mostly via common host plants or the foraging substrate such as dung (Kajtoch, 2022), the transmission pathways in Lepidoptera are unclear but could occur through feeding on common plants (Li et al., 2017; Sintupachee et al., 2006), sucking from the same mud‐pools (Duplouy et al., 2020) and exposure to ectoparasitic mites (Gehrer & Vorburger, 2012; Jaenike et al., 2007), or parasitoids (Ahmed, Li, et al., 2015; Vavre et al., 1999). Since many butterfly larvae feed on plant tissue, and adults obtain nectar from flowers or tree sap, the close association of butterflies with plants might lead to transmission being mediated through the host plant (Sintupachee et al., 2006). The mud pools could provide suitable short‐term environments for *Wolbachia* to survive and transfer to a new host niche (Duplouy et al., 2020). However, this remains to be tested (Duplouy et al., 2020). Many hymenopteran parasitoids attack lepidopteran hosts and many of these are generalist parasitoids that could mediate horizontal transmission (Apiwathnasorn, 2012).

## 5 CONCLUDING REMARKS

This study provides a conservative estimate of *Wolbachia* prevalence (14%) of the butterfly tribe Tagiadini with a species incidence of 28%, given the restricted nature of our sampling. The cophylogenetic comparison, divergence time estimation and *Wolbachia* recombination analysis all point to *Wolbachia* acquisition in Tagiadini through horizontal transmission.

## AUTHOR CONTRIBUTIONS

**Xiaoying Wei:** Investigation (supporting); methodology (lead); writing – original draft (supporting). **Jianqing Zhu:** Conceptualization (equal); resources (equal). **Ary A. Hoffmann:** Writing – review and editing (lead). **Jiqin Jia:** Investigation (equal). **Mengqi Xiao:** Investigation (equal). **Feiyu Duan:** Software (equal). **Yimin Zhang:** Software (equal). **Huimin Zhong:** Methodology (equal). **Jingyan Ge:** Methodology (equal). **Weidong Yu:** Conceptualization (equal). **Lei Zhang:** Methodology (equal). **Weibin Jiang:** Conceptualization (equal); funding acquisition (equal); writing – original draft (lead).

## FUNDING INFORMATION

We acknowledge financial support from Shanghai Natural Science Foundation (20ZR1440800), Shanghai Municipal Human Resources and Social Security Bureau (2019112) and Shanghai Normal University (SK202143).

## Supporting information


Figures S1–S2

Tables S1–S3

## Data Availability

The authors state that all data necessary for confirming the conclusions presented in the article are represented fully within the article. All original raw sequence data files are available via the GenBank (accession numbers MZ711367–MZ711409, MZ725189–MZ725317, MZ821207–MZ821249, OR479930 and OR479931).
