# Supplementary material for: Wolbachia infection status and molecular diversity in the species of tribe Tagiadini Mabille, 1878 (Lepidoptera: Hesperiidae) collected in China
Source: Ecol Evol. 2024 Apr 16;14(4):e11279. doi: 10.1002/ece3.11279 (PMC11021859; doi:10.1002/ece3.11279)
Supplement: Supplementary file 1 — Figures S1–S2 [file ECE3-14-e11279-s001.docx]

**Supplementary Figures**


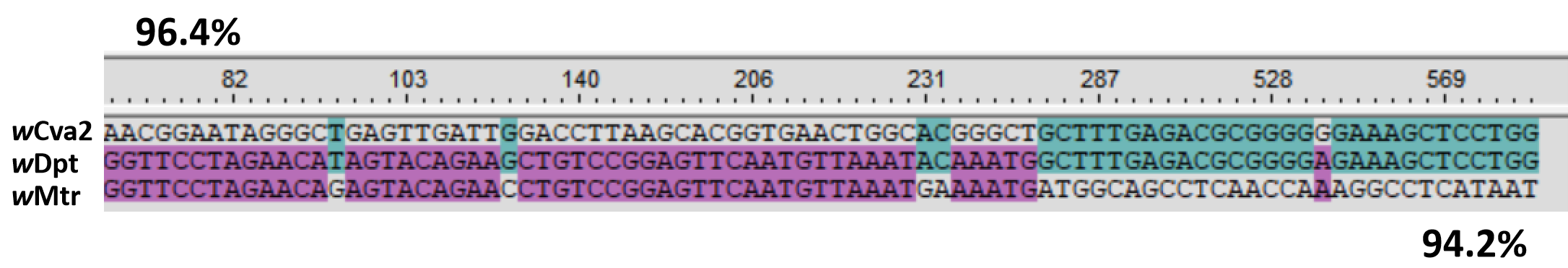


**Figure S1** Description of recombination events. For each alignment, only polymorphic sites around the breakpoints are shown. Numbers above sequence alignments indicate the schematic nucleotide position. Percentages above and under the sequence alignments show the similarities of the daughter sequence to its major and minor parent sequences (marked with the same background color).


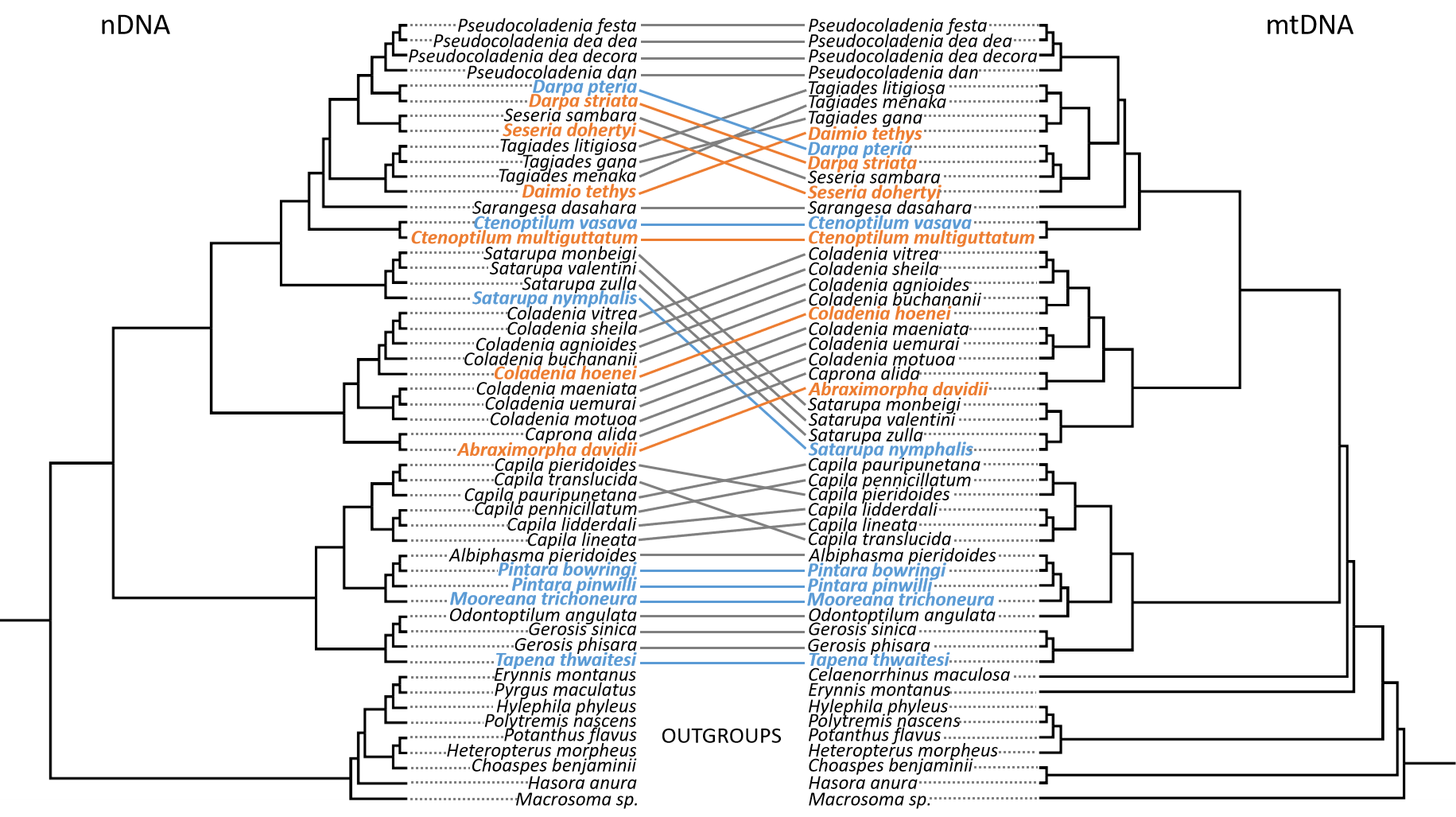


**Figure S2** Cophylogenetic analysis of Tagiadini based on mtDNA (left) and corresponding nDNA (right). Connecting lines highlight the same species. The species infected with *Wolbachia* strains of supergroup A as blue and the species infected with *Wolbachia* strains of supergroup B as orange are indicated.
